# (5*R*,6*S*)-4-Isopropyl-5-methyl-6-phenyl-3-propanoyl-2*H*-1,3,4-oxadiazinan-2-one

**DOI:** 10.1107/S1600536809022363

**Published:** 2009-06-27

**Authors:** Dishant Tailor, Kate L. Edler, David M. Casper, Shawn R. Hitchcock, Gregory M. Ferrence

**Affiliations:** a CB 4160, Department of Chemistry, Illinois State University, Normal, IL 61790, USA

## Abstract

The title compound, C_16_H_22_N_2_O_3_, was synthesized during the course of a study on (1*R*,2*S*)-norephedrine-derived 1,3,4-oxadiazinan-2-ones. The conformation adopted by the isopropyl group is pseudo-axial relative to the oxadiazinan core. The allylic strain contributes to this conformational arrangement.

## Related literature

For related structures and background, see: Casper, Blackburn *et al.* (2002 ▶); Casper, Burgeson *et al.* (2002 ▶); Casper & Hitchcock (2003 ▶); Evans *et al.* (1981 ▶); Ferrence *et al.* (2003 ▶), Hitchcock *et al.* (2001 ▶); Trepanier *et al.* (1968 ▶). The synthesis of the title compound is described by Hitchcock *et al.* (2004 ▶). For ring puckering analysis, see: Boeyens (1978 ▶); Cremer & Pople (1975 ▶); Spek (2009 ▶). For non-classical hydrogen bonding, see: Steiner (1996 ▶).
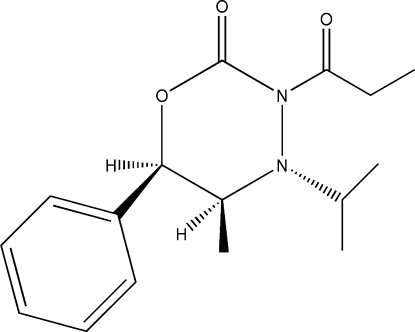


## Experimental

### 

#### Crystal data

C_16_H_22_N_2_O_3_
*M* *_r_* = 290.36
Orthorhombic, 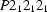

*a* = 6.8644 (3) Å
*b* = 10.8370 (5) Å
*c* = 21.2348 (10) Å
*V* = 1579.65 (12) Å^3^
*Z* = 4
Mo *K*α radiation
μ = 0.09 mm^−1^
*T* = 140 K
0.45 × 0.29 × 0.2 mm

#### Data collection

Bruker SMART APEX CCD diffractometer
Absorption correction: multi-scan (*SADABS* in *SAINT-Plus*; Bruker, 2003 ▶) *T*_min_ = 0.878, *T*_max_ = 0.983
16184 measured reflections
2263 independent reflections
2231 reflections with *I* > 2σ(*I*)
*R*_int_ = 0.022

#### Refinement

*R*[*F* ^2^ > 2σ(*F* ^2^)] = 0.039
*wR*(*F* ^2^) = 0.099
*S* = 1.18
2263 reflections
190 parameters
H-atom parameters constrained
Δρ_max_ = 0.32 e Å^−3^
Δρ_min_ = −0.21 e Å^−3^

### 

Data collection: *SMART* (Bruker, 2003 ▶); cell refinement: *SAINT-Plus* (Bruker, 2003 ▶); data reduction: *SAINT-Plus*; program(s) used to solve structure: *SIR2004* (Burla *et al.*, 2005 ▶); program(s) used to refine structure: *SHELXL97* (Sheldrick, 2008 ▶); molecular graphics: *ORTEP-3 for Windows* (Farrugia, 1997 ▶); software used to prepare material for publication: *WinGX* (Farrugia, 1999 ▶) and *publCIF* (Westrip, 2009 ▶).

## Supplementary Material

Crystal structure: contains datablocks global, I. DOI: 10.1107/S1600536809022363/zl2203sup1.cif

Structure factors: contains datablocks I. DOI: 10.1107/S1600536809022363/zl2203Isup2.hkl

Additional supplementary materials:  crystallographic information; 3D view; checkCIF report

**Fig. 2.**
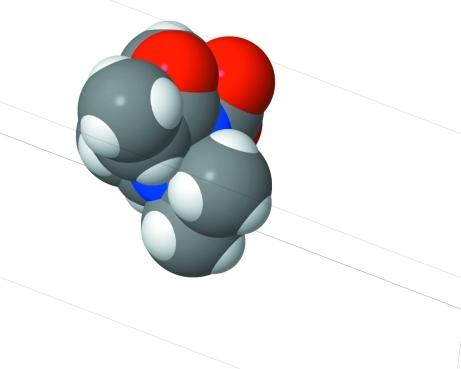
J mol enhanced figure of the title compound. The default view shows a space-filling depiction of the asymmetric unit. Key torsion angles may be highlighted when viewing the active enhanced figure.

Enhanced figure: interactive version of Fig. 2

## Figures and Tables

**Table 1:** Hydrogen-bond geometry (Å, °)

*D*—H⋯*A*	*D*—H	H⋯*A*	*D*⋯*A*	*D*—H⋯*A*
C8—H8⋯O1	0.95	2.40	2.737 (2)	101
C14—H14⋯O1	1.00	2.55	3.091 (2)	114
C15—H15*A*⋯N3	0.98	2.50	2.839 (2)	100
C5—H5⋯O20^i^	1.00	2.42	3.263 (2)	142
C16—H16*B*⋯O21^i^	0.98	2.40	3.380 (3)	175
C12—H12⋯O21^i^	0.95	2.35	3.243 (2)	156
C5—H5⋯O21^i^	1.00	2.40	3.283 (2)	147
C6—H6⋯O1^ii^	1.00	2.58	3.543 (2)	163

Symmetry codes: (i) 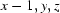; (ii) 
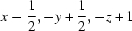
.

**Fig. 1.**
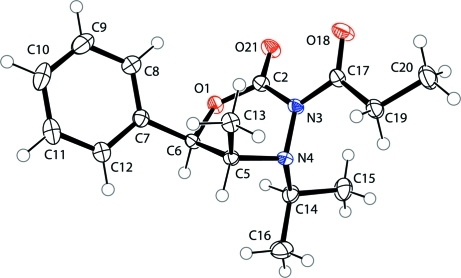
The molecular structure of the title compound, with the atomic numbering scheme. Displacement ellipsoids are drawn at the 50% probability level.

## supplementary crystallographic information

### Comment

The production of enantiomerically pure compounds has become increasingly important in the pharmaceutical industry. The high demand for a single enantiomer of a chiral intermediate has led to a wealth of methods for asymmetric synthesis (Hitchcock *et al.*, 2004). While asymmetric catalysis and other methods have been functional in asymmetric synthesis, the important role of chiral auxiliaries in asymmetric synthesis is patent. Oxazolidin-2-ones, chiral auxiliaries first introduced by Evans *et al.* (Evans *et al.*, 1981), have been of substance in areas of alkylation reactions, pericyclic reactions, and asymmetric aldol condensation reactions. Related, 1,3,4-oxadiazinan-2-one heterocycles have received little interest since their disclosure (Trepanier *et al.*, 1968). It was not until recently that synthetic (Hitchcock *et al.*, 2001) and conformational studies (Casper, Burgeson *et al.*, 2002) of 1,3,4-oxadiazinan-2-one have been thoroughly performed.

Herein we report the X-ray structure of the N3-propanoyl acylated norephedrine-derived 1,3,4-oxadiazinan-2-one. The imide carbonyls adopt a *syn*-periplanar orientation, with an O21—C2—C17—O20 torsion angle of 23.67 (17)°. This result is consistent with those of previously reported N3 substituted oxadiazinan-2-ones (Casper, Blackburn *et al.*, 2002; Casper, Burgeson *et al.*, 2002; Ferrence *et al.*, 2003). It is believed that in the oxadiazinaneone systems the *syn*-periplanar conformation arises from the lone pair repulsion interaction between the N4-nitrogen lone pair and the N3-carbonyl lone pair (Casper, Blackburn *et al.*, 2002). However, in the case of the title compound, the repulsive interactions between the N3-substituent and the N4-isopropyl could also be held accountable for the *syn*-periplanar orientation. In fact, ring puckering analysis using *PLATON* (Spek, 2009; Cremer & Pople, 1975; Boeyens, 1978) indicates θ = 62.7 (2)° and Φ = 196.9 (2)° for the O1—C2—N3—N4—C5—C6 ring, which is consistent with a formal conformational assignment close to an idealized E_4_ envelope with N4 being the flap apex. Such a conformation possesses a pseudo-axial C5-methyl group, a typical pseudo-equatorial C6-phenyl ring, and a typical pseudo-axial N4-iso-propyl group. The imide carbonyls, although not *syn*-parallel, indicate the existence of resonance delocalization due to their approximately planar conformation [torsion angle 23.67 (17)°]. Based on previous studies (Casper, Blackburn *et al.*, 2002), the N3-substituent is held rigidly due to resonance interactions, while the N4-isopropyl group adopts a pseudoaxial orientation to relieve allylic strain on the system. Both intra- and inter-molecular non-classical H-bonding interactions exist. Those interactions shorter than 2.7 Å with a >90° D—H···A angle are shown in Table 1 (Steiner, 1996). It appears that such non-classical H-bonding interactions may constitute the dominant packing forces in this structure; however, evaluation of additional related structures will be necessary before any particular rational for these interactions is defensible.

### Experimental

The title compound was synthesized by acylation of norephedrine derived 1,3,4-oxadiazinan-2-one using propanoyl (Hitchcock *et al.*, 2004). Single crystals were grown by vapor diffusion of hexane into a methylene chloride solution of the title compound.

### Refinement

All non-H atoms were refined anisotropically without disorder. All H atoms were initially identified through difference Fourier syntheses and then removed and included in the refinement in the riding-model approximation with fixed individual displacement parameters [U(H_iso_) = 1.2*U*_eq_(C) or U(H_iso_) = 1.5*U*_eq_(C_methyl_)] using a riding model with C_aromatic_—H = 0.95 Å, C_methyl_—H = 0.98 Å, C_methylene_—H = 0.99 Å or C_methine_—H = 1.00 Å. Friedel opposites were merged.

### Crystal data

**Table:**

C_16_H_22_N_2_O_3_	*F*(000) = 624
*M**_r_* = 290.36	*D*_x_ = 1.221 Mg m^−^^3^
Orthorhombic, *P*2_1_2_1_2_1_	Mo *K*α radiation, λ = 0.71073 Å
Hall symbol: P 2ac 2ab	Cell parameters from 7989 reflections
*a* = 6.8644 (3) Å	θ = 2.7–30.4°
*b* = 10.8370 (5) Å	µ = 0.09 mm^−^^1^
*c* = 21.2348 (10) Å	*T* = 140 K
*V* = 1579.65 (12) Å^3^	Needle, colourless
*Z* = 4	0.45 × 0.29 × 0.2 mm

### Data collection

**Table:**

Bruker SMART APEX CCD diffractometer	2263 independent reflections
Radiation source: sealed tube	2231 reflections with *I* > 2σ(*I*)
graphite	*R*_int_ = 0.022
ω scans	θ_max_ = 28.3°, θ_min_ = 1.9°
Absorption correction: multi-scan (*SADABS* in *SAINT-Plus*; Bruker, 2003)	*h* = −9→9
*T*_min_ = 0.878, *T*_max_ = 0.983	*k* = −14→14
16184 measured reflections	*l* = −28→27

### Refinement

**Table:**

Refinement on *F*^2^	0 restraints
Least-squares matrix: full	H-atom parameters constrained
*R*[*F*^2^ > 2σ(*F*^2^)] = 0.039	*w* = 1/[σ^2^(*F*_o_^2^) + (0.0467*P*)^2^ + 0.4577*P*] where *P* = (*F*_o_^2^ + 2*F*_c_^2^)/3
*wR*(*F*^2^) = 0.099	(Δ/σ)_max_ < 0.001
*S* = 1.18	Δρ_max_ = 0.32 e Å^−^^3^
2263 reflections	Δρ_min_ = −0.21 e Å^−^^3^
190 parameters	

### Special details

**Table:**

Geometry. All e.s.d.'s (except the e.s.d. in the dihedral angle between two l.s. planes) are estimated using the full covariance matrix. The cell e.s.d.'s are taken into account individually in the estimation of e.s.d.'s in distances, angles and torsion angles; correlations between e.s.d.'s in cell parameters are only used when they are defined by crystal symmetry. An approximate (isotropic) treatment of cell e.s.d.'s is used for estimating e.s.d.'s involving l.s. planes.

### Fractional atomic coordinates and isotropic or equivalent isotropic displacement parameters (Å^2^)

**Table:**

	*x*	*y*	*z*	*U*_iso_*/*U*_eq_	
O1	0.75439 (18)	0.26491 (11)	0.44189 (6)	0.0196 (3)	
C2	0.8863 (3)	0.34214 (16)	0.41773 (8)	0.0188 (3)	
N3	0.8136 (2)	0.44240 (13)	0.38381 (7)	0.0178 (3)	
N4	0.6120 (2)	0.47290 (13)	0.39003 (6)	0.0166 (3)	
C5	0.5006 (2)	0.36039 (15)	0.37440 (7)	0.0165 (3)	
H5	0.3593	0.3803	0.3796	0.020*	
C6	0.5509 (2)	0.25838 (16)	0.42207 (8)	0.0163 (3)	
H6	0.4683	0.2717	0.4602	0.020*	
C7	0.5138 (3)	0.12801 (16)	0.39890 (8)	0.0187 (3)	
C8	0.6638 (3)	0.05224 (17)	0.37881 (9)	0.0262 (4)	
H8	0.7946	0.0806	0.3807	0.031*	
C9	0.6234 (4)	−0.06520 (19)	0.35596 (10)	0.0326 (5)	
H9	0.7270	−0.1166	0.3421	0.039*	
C10	0.4342 (4)	−0.10773 (17)	0.35327 (9)	0.0320 (5)	
H10	0.4073	−0.1883	0.3380	0.038*	
C11	0.2841 (3)	−0.03214 (19)	0.37300 (10)	0.0315 (4)	
H11	0.1535	−0.0608	0.3710	0.038*	
C12	0.3230 (3)	0.08577 (17)	0.39586 (9)	0.0251 (4)	
H12	0.2191	0.1373	0.4094	0.030*	
C13	0.5331 (3)	0.32666 (16)	0.30550 (8)	0.0208 (3)	
H13A	0.4971	0.3968	0.2788	0.031*	
H13B	0.4523	0.2552	0.2946	0.031*	
H13C	0.6706	0.3063	0.2988	0.031*	
C14	0.5736 (3)	0.52506 (17)	0.45405 (8)	0.0225 (4)	
H14	0.6017	0.4607	0.4865	0.027*	
C15	0.7025 (3)	0.63686 (18)	0.46590 (10)	0.0310 (4)	
H15A	0.8396	0.6123	0.4632	0.046*	
H15B	0.6756	0.6700	0.5079	0.046*	
H15C	0.6752	0.7002	0.4342	0.046*	
C16	0.3600 (3)	0.5623 (2)	0.45843 (11)	0.0344 (5)	
H16B	0.2776	0.4899	0.4511	0.052*	
H16A	0.3318	0.6253	0.4266	0.052*	
H16C	0.3334	0.5958	0.5004	0.052*	
C17	0.9228 (2)	0.50008 (16)	0.33572 (8)	0.0195 (3)	
C18	0.8239 (3)	0.60532 (17)	0.30136 (9)	0.0245 (4)	
H18A	0.7224	0.5718	0.2730	0.029*	
H18B	0.7594	0.6601	0.3323	0.029*	
C19	0.9691 (3)	0.6793 (2)	0.26320 (11)	0.0344 (5)	
H19C	0.9013	0.7465	0.2414	0.052*	
H19B	1.0317	0.6254	0.2322	0.052*	
H19A	1.0682	0.7139	0.2913	0.052*	
O20	1.08450 (19)	0.46487 (14)	0.32269 (7)	0.0296 (3)	
O21	1.05596 (18)	0.32428 (13)	0.42830 (6)	0.0266 (3)	

### Atomic displacement parameters (Å^2^)

**Table:**

	*U*^11^	*U*^22^	*U*^33^	*U*^12^	*U*^13^	*U*^23^
O1	0.0168 (6)	0.0205 (6)	0.0214 (6)	−0.0011 (5)	−0.0033 (5)	0.0057 (5)
C2	0.0173 (7)	0.0213 (8)	0.0177 (7)	0.0005 (7)	−0.0004 (6)	0.0014 (6)
N3	0.0122 (6)	0.0190 (6)	0.0222 (7)	0.0004 (6)	0.0001 (5)	0.0033 (5)
N4	0.0121 (6)	0.0184 (6)	0.0194 (6)	0.0011 (5)	0.0013 (5)	−0.0003 (5)
C5	0.0144 (7)	0.0168 (7)	0.0183 (7)	0.0007 (6)	−0.0004 (6)	0.0017 (6)
C6	0.0126 (7)	0.0188 (7)	0.0174 (7)	−0.0009 (6)	0.0006 (6)	0.0014 (6)
C7	0.0216 (8)	0.0183 (7)	0.0163 (7)	−0.0009 (7)	0.0006 (6)	0.0015 (6)
C8	0.0260 (9)	0.0229 (8)	0.0297 (9)	0.0018 (8)	0.0019 (8)	−0.0018 (7)
C9	0.0420 (12)	0.0240 (9)	0.0318 (10)	0.0069 (9)	0.0038 (9)	−0.0036 (8)
C10	0.0539 (13)	0.0182 (8)	0.0239 (9)	−0.0074 (9)	−0.0026 (9)	−0.0011 (7)
C11	0.0337 (10)	0.0287 (10)	0.0322 (10)	−0.0126 (9)	−0.0017 (9)	0.0020 (8)
C12	0.0238 (9)	0.0230 (8)	0.0287 (9)	−0.0032 (8)	0.0008 (8)	0.0002 (7)
C13	0.0223 (8)	0.0226 (8)	0.0176 (7)	−0.0019 (8)	−0.0014 (6)	0.0010 (6)
C14	0.0281 (9)	0.0190 (7)	0.0203 (8)	−0.0011 (7)	0.0038 (7)	−0.0027 (6)
C15	0.0372 (11)	0.0242 (9)	0.0315 (9)	−0.0071 (8)	−0.0018 (9)	−0.0061 (8)
C16	0.0311 (10)	0.0337 (10)	0.0384 (11)	0.0030 (9)	0.0099 (9)	−0.0119 (9)
C17	0.0172 (7)	0.0208 (8)	0.0206 (7)	−0.0032 (7)	0.0000 (6)	0.0024 (6)
C18	0.0202 (8)	0.0239 (8)	0.0295 (9)	0.0003 (8)	0.0032 (7)	0.0086 (7)
C19	0.0281 (10)	0.0326 (10)	0.0424 (11)	−0.0031 (9)	0.0066 (9)	0.0167 (9)
O20	0.0183 (6)	0.0387 (8)	0.0318 (7)	0.0052 (6)	0.0061 (5)	0.0110 (6)
O21	0.0160 (6)	0.0325 (7)	0.0314 (7)	0.0020 (6)	−0.0018 (5)	0.0102 (6)

### Geometric parameters (Å, °)

**Table:**

O1—C2	1.336 (2)	C11—H11	0.9500
O1—C6	1.460 (2)	C12—H12	0.9500
C2—O21	1.202 (2)	C13—H13A	0.9800
C2—N3	1.396 (2)	C13—H13B	0.9800
N3—C17	1.413 (2)	C13—H13C	0.9800
N3—N4	1.4291 (19)	C14—C15	1.521 (3)
N4—C5	1.477 (2)	C14—C16	1.523 (3)
N4—C14	1.496 (2)	C14—H14	1.0000
C5—C13	1.524 (2)	C15—H15A	0.9800
C5—C6	1.538 (2)	C15—H15B	0.9800
C5—H5	1.0000	C15—H15C	0.9800
C6—C7	1.518 (2)	C16—H16B	0.9800
C6—H6	1.0000	C16—H16A	0.9800
C7—C8	1.384 (3)	C16—H16C	0.9800
C7—C12	1.389 (3)	C17—O20	1.206 (2)
C8—C9	1.390 (3)	C17—C18	1.515 (2)
C8—H8	0.9500	C18—C19	1.514 (3)
C9—C10	1.379 (3)	C18—H18A	0.9900
C9—H9	0.9500	C18—H18B	0.9900
C10—C11	1.381 (3)	C19—H19C	0.9800
C10—H10	0.9500	C19—H19B	0.9800
C11—C12	1.393 (3)	C19—H19A	0.9800
			
C2—O1—C6	124.62 (13)	C5—C13—H13A	109.5
O21—C2—O1	118.98 (16)	C5—C13—H13B	109.5
O21—C2—N3	124.62 (16)	H13A—C13—H13B	109.5
O1—C2—N3	116.34 (15)	C5—C13—H13C	109.5
C2—N3—C17	121.86 (14)	H13A—C13—H13C	109.5
C2—N3—N4	118.59 (14)	H13B—C13—H13C	109.5
C17—N3—N4	118.58 (14)	N4—C14—C15	110.40 (15)
N3—N4—C5	106.83 (12)	N4—C14—C16	108.99 (15)
N3—N4—C14	110.01 (13)	C15—C14—C16	109.78 (16)
C5—N4—C14	115.14 (13)	N4—C14—H14	109.2
N4—C5—C13	109.75 (13)	C15—C14—H14	109.2
N4—C5—C6	109.23 (13)	C16—C14—H14	109.2
C13—C5—C6	115.24 (14)	C14—C15—H15A	109.5
N4—C5—H5	107.4	C14—C15—H15B	109.5
C13—C5—H5	107.4	H15A—C15—H15B	109.5
C6—C5—H5	107.4	C14—C15—H15C	109.5
O1—C6—C7	107.41 (14)	H15A—C15—H15C	109.5
O1—C6—C5	111.69 (13)	H15B—C15—H15C	109.5
C7—C6—C5	114.71 (13)	C14—C16—H16B	109.5
O1—C6—H6	107.6	C14—C16—H16A	109.5
C7—C6—H6	107.6	H16B—C16—H16A	109.5
C5—C6—H6	107.6	C14—C16—H16C	109.5
C8—C7—C12	119.44 (17)	H16B—C16—H16C	109.5
C8—C7—C6	121.81 (16)	H16A—C16—H16C	109.5
C12—C7—C6	118.71 (15)	O20—C17—N3	120.94 (16)
C7—C8—C9	120.16 (19)	O20—C17—C18	122.72 (16)
C7—C8—H8	119.9	N3—C17—C18	116.33 (15)
C9—C8—H8	119.9	C19—C18—C17	111.18 (16)
C10—C9—C8	120.54 (19)	C19—C18—H18A	109.4
C10—C9—H9	119.7	C17—C18—H18A	109.4
C8—C9—H9	119.7	C19—C18—H18B	109.4
C9—C10—C11	119.44 (18)	C17—C18—H18B	109.4
C9—C10—H10	120.3	H18A—C18—H18B	108.0
C11—C10—H10	120.3	C18—C19—H19C	109.5
C10—C11—C12	120.46 (19)	C18—C19—H19B	109.5
C10—C11—H11	119.8	H19C—C19—H19B	109.5
C12—C11—H11	119.8	C18—C19—H19A	109.5
C7—C12—C11	119.96 (18)	H19C—C19—H19A	109.5
C7—C12—H12	120.0	H19B—C19—H19A	109.5
C11—C12—H12	120.0		
			
C6—O1—C2—O21	−165.68 (17)	O1—C6—C7—C12	160.20 (15)
C6—O1—C2—N3	17.1 (2)	C5—C6—C7—C12	−75.0 (2)
O21—C2—N3—C17	29.6 (3)	C12—C7—C8—C9	−0.2 (3)
O1—C2—N3—C17	−153.40 (15)	C6—C7—C8—C9	−177.88 (17)
O21—C2—N3—N4	−161.87 (17)	C7—C8—C9—C10	−0.3 (3)
O1—C2—N3—N4	15.2 (2)	C8—C9—C10—C11	0.6 (3)
C2—N3—N4—C5	−55.15 (18)	C9—C10—C11—C12	−0.4 (3)
C17—N3—N4—C5	113.79 (15)	C8—C7—C12—C11	0.3 (3)
C2—N3—N4—C14	70.48 (18)	C6—C7—C12—C11	178.08 (16)
C17—N3—N4—C14	−120.58 (16)	C10—C11—C12—C7	0.0 (3)
N3—N4—C5—C13	−64.81 (16)	N3—N4—C14—C15	56.73 (18)
C14—N4—C5—C13	172.72 (14)	C5—N4—C14—C15	177.48 (15)
N3—N4—C5—C6	62.43 (16)	N3—N4—C14—C16	177.38 (15)
C14—N4—C5—C6	−60.04 (17)	C5—N4—C14—C16	−61.87 (18)
C2—O1—C6—C7	120.86 (16)	C2—N3—C17—O20	−0.7 (3)
C2—O1—C6—C5	−5.7 (2)	N4—N3—C17—O20	−169.21 (16)
N4—C5—C6—O1	−34.68 (18)	C2—N3—C17—C18	178.17 (16)
C13—C5—C6—O1	89.39 (17)	N4—N3—C17—C18	9.6 (2)
N4—C5—C6—C7	−157.20 (14)	O20—C17—C18—C19	−15.4 (3)
C13—C5—C6—C7	−33.1 (2)	N3—C17—C18—C19	165.80 (17)
O1—C6—C7—C8	−22.1 (2)	O21—C2—C17—O20	23.67 (17)
C5—C6—C7—C8	102.74 (19)		

### Hydrogen-bond geometry (Å, °)

**Table:**

*D*—H···*A*	*D*—H	H···*A*	*D*···*A*	*D*—H···*A*
C8—H8···O1	0.95	2.40	2.737 (2)	101
C14—H14···O1	1.00	2.55	3.091 (2)	114
C15—H15A···N3	0.98	2.50	2.839 (2)	100
C5—H5···O20^i^	1.00	2.42	3.263 (2)	142
C16—H16B···O21^i^	0.98	2.40	3.380 (3)	175
C12—H12···O21^i^	0.95	2.35	3.243 (2)	156
C5—H5···O21^i^	1.00	2.40	3.283 (2)	147
C6—H6···O1^ii^	1.00	2.58	3.543 (2)	163

Symmetry codes: (i) *x*−1, *y*, *z*; (ii) *x*−1/2, −*y*+1/2, −*z*+1.
